# Worst Pattern of Perineural Invasion Redefines the Spatial Localization of Nerves in Oral Squamous Cell Carcinoma

**DOI:** 10.3389/fonc.2021.766902

**Published:** 2021-11-29

**Authors:** Yong Fu, Xinwen Zhang, Zhuang Ding, Nisha Zhu, Yuxian Song, Xiaoxin Zhang, Yue Jing, Yijun Yu, Xiaofeng Huang, Lei Zhang, Qingang Hu, Yanhong Ni, Liang Ding

**Affiliations:** ^1^ Central Laboratory of Stomatology, Nanjing Stomatological Hospital, Medical School of Nanjing University, Nanjing, China; ^2^ Department of Oral Pathology, Nanjing Stomatological Hospital, Medical School of Nanjing University, Nanjing, China

**Keywords:** tumor-nerve interaction, worst pattern of perineural invasion (WPNI), oral squamous cell carcinoma (OSCC), prognostic biomarker, immune balance

## Abstract

As a key histopathological characteristic of tumor invasion, perineural invasion (PNI) assists tumor dissemination, whereas the current definition of PNI by dichotomy is not accurate and the prognostic value of PNI has not reached consensus. To define PNI status in each patient when mixed types of PNI occurred simultaneously, we here further subclassified the traditional PNI in 183 patients with oral squamous cell carcinoma (OSCC). The spatial localization of nerves in OSCC microenvironment was thoroughly evaluated and successfully concluded into four types of PNI: 0, tumor cells away from nerves; 1, tumor cells encircling nerves less than 33%; 2, tumor cells encircling nerves at least 33%; and 3, tumor cells infiltrating into nerve sheathes. Sequentially, patients were stratified by single and mixed types of PNI. Traditionally, types 0 and 1 were defined as PNI^−^, while types 2 and 3 were PNI^+^, which predicted shorter survival time. When multiple types of PNI existed within one tumor, patients with higher score of PNI types tended to have a relatively worse prognosis. Therefore, to define the status of PNI more precisely, the new variable worst pattern of PNI (WPNI) was proposed, which was taken as the highest score of PNI types present in each patient no matter how focal. Results showed that patients with WPNI 1 had longest survival time, and WPNI 2 correlated with better overall survival (*p* = 0.02), local-regional recurrence-free survival (*p* = 0.03), and distant metastasis-free survival (*p* = 0.046) than WPNI 3. Multivariate Cox analysis confirmed that only WPNI 3 could independently predict patients’ prognosis, which could be explained by a more damaged immune response in WPNI 3 patients with less CD3^+^CD8^+^ T cells and CD19^+^ B cells. Conclusively, WPNI by trichotomy provide more meticulous and precise pathological information for tumor-nerve interactions in OSCC patients.

## Introduction

Oral squamous cell carcinoma (OSCC) represents the most common epithelial malignancy in the head and region, with nearly 350,000 new cases and 180,000 deaths in 2018 globally (1). Smoking, alcohol drinking, and betel quid chewing are reported as lifestyle-related pathogenic risk factors (2). At present, the primary choice for OSCC treatment is still surgical resection supplemented with/without postoperative radiotherapy, chemotherapy, or concurrent chemoradiotherapy (3). Although strategies for OSCC diagnosis and treatment are both constantly being optimized, the 5-year overall survival rate remains about 60% (3). With OSCC progression, patients eventually die of tumor recurrence and metastasis (2). Typically, lymphovascular route represents the main pathway for OSCC metastasis while nerves as the potential pathway have attracted increasing attentions in recent years (4).

In the tumor microenvironment, nerves have been neglected by researchers (5). However, in addition to interacting with immune cells, fibroblasts, and endothelial cells, tumor cells can also interact with nerves (6). Morphologically, the best example of tumor-nerve interaction is perineural invasion (PNI), which assists tumor dissemination and has been recognized as a negative prognostic factor for several cancers (7–10). Although PNI is included in clinical diagnosis including OSCC (11), there is still lack of a standardized definition consensus among pathologists (12, 13). In 1985, Batsakis et al. described PNI as tumor cells invaded in, around, and through peripheral nerves (14), nerves were surrounded by tumor cells in whole or in part, or tumor cells were observed inside the endoneurium (15). With traditional dichotomies, PNI was classified as positive (presence, PNI^+^) or negative (absence, PNI^−^).

However, debates on the spatial relationship between tumor cells and nerves in PNI have existed for decades due to this dichotomy. On the one hand, Liebig et al. optimized PNI to the definition that tumor cells are closely adjacent to the peripheral nerves and encircle no less than 33% of their circumferences or tumor cells within any of the three layers of nerve sheaths; however, the judgement of PNI in clinical practice was still quite subjective (13, 15, 16). Consequently, the detection rate of positive PNI in the same tumor type varied greatly across cohorts (4). Additionally, a few studies still argued that PNI failed to predict survival (17–20). However, on another hand, intratumoral heterogeneity within a single tumor microenvironment (TME) is the intrinsic driver for the simultaneous existence of several PNI types. The status of PNI in each patient as mixed types of PNI coexist and their clinical outcomes are unclear, which promoted us to identify a histopathological indicator to efficiently capture the feature of tumor-nerve interaction patterns.

In this study, in order to further classify the traditional PNI, we thoroughly evaluated the spatial localization of nerves in OSCC microenvironment. Then, the new variable, the worst pattern of PNI (WPNI) was proposed and investigated for its clinical significance. Moreover, as increasing evidence suggests that peripheral nerves profoundly alter the immune response in both inflammatory diseases and cancers (21–23), we also explored whether the imbalance of the immune system was associated with different WPNI scores in OSCC patients.

## Materials and Methods

### Patients and Tissue Samples

A total of 183 patients with primary OSCC treated in the Department of Oral and Maxillofacial Surgery at Nanjing Stomatological Hospital from January 2013 to December 2014 were included in this retrospective study (**Table 1**). Patients’ demographic data (age and sex), clinicopathological parameters (tumor site, pathologic T stage, pathologic N stage, pathologic TNM stage, tumor differentiation, worst pattern of invasion (WPOI), PNI, local-regional control and distant metastasis), and treatment modalities (radiotherapy and chemotherapy) were included and analyzed. Inclusion criteria included the following: (1) patients with a pathological diagnosis of OSCC; (2) patients who were primarily treated with surgery; and (3) patients with complete clinicopathological data and available tissue specimens. The exclusion criteria included preoperative chemotherapy or radiotherapy, failure to undergo surgery, and the inability to obtain pathological slides. The pathological stages and the histological grade of OSCC were separately classified based on the guidelines of the 7th edition of AJCC Cancer Staging and the protocol of WHO. This study was conducted in full accordance with ethical principles and was approved by the Medical Ethics Committee of the Nanjing Stomatological Hospital, Medical School of Nanjing University [approval number: 2019NL-009(KS)].

**Table 1:** Clinicopathological features of 183 OSCC patients in this study.

Features	*n* (%)
Age (years): median (range)	61 (26–83)
Follow-up (months): median (range)	73 (2–87)
Sex	
Female	78 (42.6)
Male	105 (57.4)
Tumor site	
Buccal mucosa	33 (18.0)
Tongue	84 (45.9)
Gingiva	33 (18.0)
Others	33 (18.0)
Pathologic T stage	
T1	66 (36.1)
T2	91 (49.7)
T3	14 (7.7)
T4	12 (6.6)
Pathologic N stage	
N0	118 (64.5)
N1	38 (20.8)
N2	27 (14.8)
Tumor TNM stage	
I	46 (25.1)
II	57 (31.1)
III	45 (24.6)
IV	35 (19.1)
Tumor differentiation	
Well	163 (89.1)
Moderately/Poor	20 (10.9)
Worst pattern of invasion (WPOI)	
1–3	95 (51.9)
4–5	88 (48.1)
Radiotherapy	
Without	114 (62.3)
With	69 (37.7)
Chemotherapy	
Without	159 (86.9)
With	24 (13.1)

TNM, tumor-node-metastasis.

### Study Design

**Figure 1:**
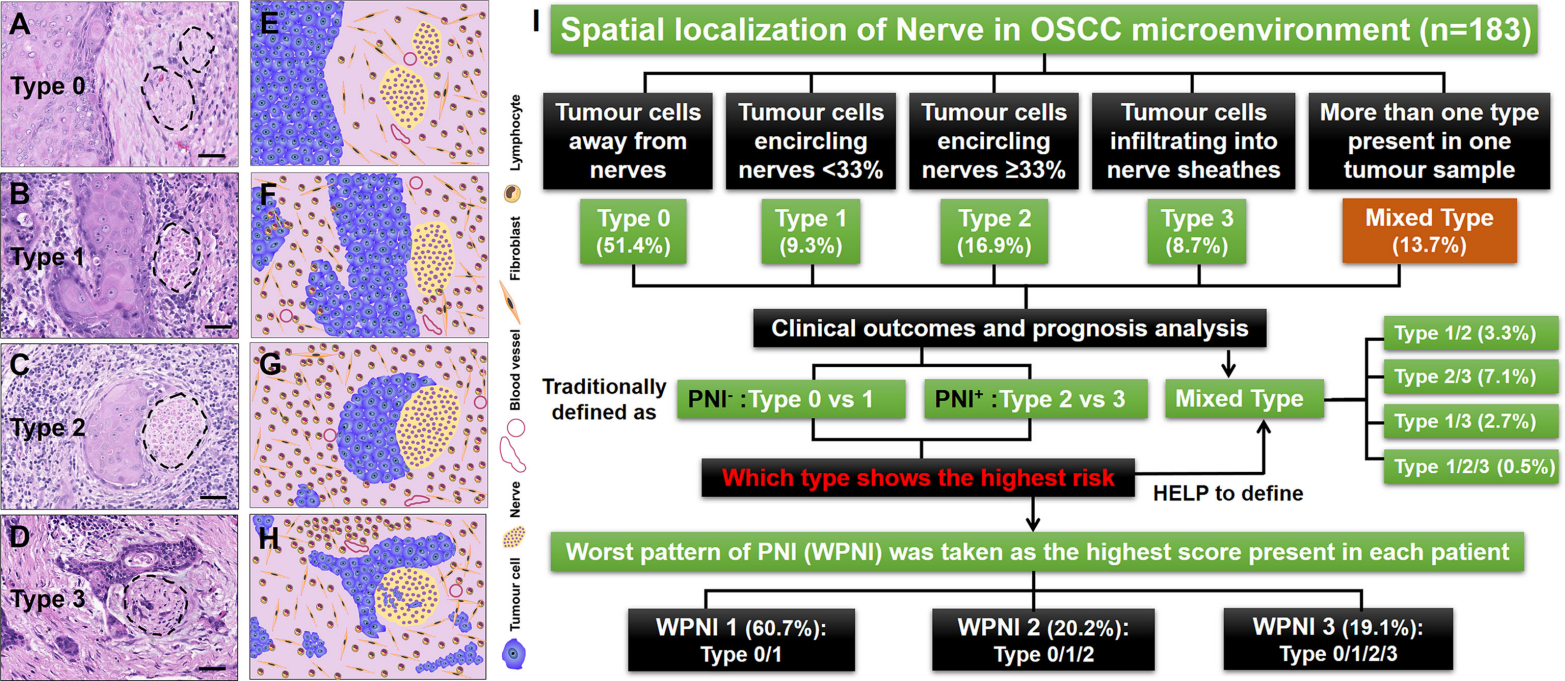
Illustrations for patterns of tumor-nerve interaction and the study design. **(A)** PNI type 0, tumor cells away from nerves. **(B)** PNI type 1, tumor cells encircling nerves less than 33%. **(C)** PNI type 2, tumor cells encircling nerves at least 33%. **(D)** PNI type 3, tumor cells infiltrating into nerve sheathes. **(E**–**H)** The cartoon diagrams corresponding to **(A**–**D)**. **(I)** Flow diagram illustrating the management of OSCC patients stratified by different types of PNI. The dashed circles represented the peripheral nerves. Scale bars: 20 μm.

Based on distinct spatial localization of nerves in OSCC microenvironment, 183 OSCC patients were stratified by five types of PNI as indicated in **Figure 1I**. Through evaluation of their prognostic value, we tried to determine which type of PNI showed the highest risk of death, especially when mixed types of PNI occurred in one patient. Finally, we introduced the new variable worst pattern of PNI (WPNI) to define the PNI status of OSCC patients and further evaluated its predictive ability for the clinical outcome.

### PNI Types and the WPNI

The traditional PNI were subclassified into four types: 0, tumor cells away from nerves; 1, tumor cells encircling nerves less than 33%; 2, tumor cells encircling nerves at least 33%; and 3, tumor cells infiltrating into nerve sheathes. The new variable WPNI was taken as the highest score of PNI types present in each patient no matter how focal, which was similar to WPOI (24). Two oral pathologists independently reviewed all hematoxylin-eosin (H&E)-stained slides to recognize and record the existing PNI types and where disagreement occurred, consensus would be reached through a discussion.

### Immunohistochemistry

Immunohistochemical staining was performed on 4-μm-thick formalin-fixed, paraffin-embedded tissue sections. After baking all sections at 70°C for 45 min, they were incubated with xylene three times for 10 min each and then treated with gradient ethanol for 5 min in each solution. Antigen unmasking was performed by boiling the sections in sodium citrate buffer (pH 6.0), blocking with 3% hydrogen peroxide for 10 min at room temperature and washing. Cytokeratin-5/6 (kit-0018) and S-100β (kit-0007), both ready-to-use and purchased from Maixin (Maixin Biotech Co., Ltd., Fuzhou, China), were used to label tumor epithelial cells and nerves, respectively, at 4°C overnight. Then, the Super-MaxVision mouse/rabbit Universal HRP Kit (TPB-0015, Typing Biotech Co., Ltd., Nanjing, China) was used for DAB chromogen staining followed by nuclear staining using hematoxylin. Sections were covered with neutral gum and dried at room temperature.

### Flow Cytometry

The T/B/NK cells data in preoperative blood of primary OSCC patients was immediately collected and analyzed using flow cytometry. To identify and determine the percentages of mature human lymphocyte subsets in erythrocyte-lysed whole blood, including T cells (CD3^+^), B cells (CD19^+^), helper/inducer T cells (CD3^+^CD4^+^), suppressor/cytotoxic T cells (CD3^+^CD8^+^), and natural killer (NK) lymphocytes (CD3^−^CD16^+^ and/or CD56^+^), BD Multitest™ CD3-FITC/CD8-PE/CD45-PerCP/CD4-APC reagent and BD Multitest™ CD3-FITC/CD16-PE+CD56-PE/CD45-PerCP/CD19-APC reagent were used according to the manufacturer’s instructions (Cat No. 340503, BD Biosciences, Franklin Lakes, NJ, USA), and samples were then quantified by flow cytometry on a FACS Calibur instrument. To determine the absolute counts of the lymphocyte subsets listed above, the total numbers of preoperative peripheral lymphocytes determined by the Automated Haematology Analyser XS Series (XS-1000i, Sysmex Corporation, Japan) were collected from the clinical laboratory. Since both tests came from the same batch of blood samples, we ignored the possible errors caused by the use of different detection instruments. Herein, the TBNK data of 62.3% (114/183) of OSCC patients were successfully collected and the detailed characteristic data are listed in the **Supplementary Table S1**.

### Statistical Analysis

Survival curves were calculated by the Kaplan-Meier method and compared by the log-rank test. The hazard ratio (HR) was calculated using the Cox proportional hazard regression model. Overall survival (OS) was defined as the time from surgery to death from any cause. Local-regional recurrence-free survival (LRFS) and distant metastasis-free survival (DMFS) were defined as the time from surgery to the occurrence of local-regional recurrence and distant metastasis or death from any cause, respectively.

For descriptive analysis, categorical variables are expressed as numbers and percentages, and continuous variables are expressed as median values and ranges. The Chi-square test was used to compare the correlations between the baseline factors and the morphological classifications of PNI. All hypothesis generation tests were two sided, and differences between groups were analyzed using Student’s *t*-test, with a significance level of 0.05: ^*^ *p* < 0.05; ^**^ *p* < 0.01; and ^***^ *p* < 0.001. Data analysis and visualization were performed on the Windows platform using IBM SPSS 24.0 and GraphPad Prism 8.0.

## Results

### OSCC Microenvironment Has Heterogeneous Patterns of Tumor-Nerve Interaction

A total of 183 patients with primary OSCC were enrolled in this study, and 904 H&E-stained slides were thoroughly reviewed. Based on the spatial localization of nerves in the OSCC microenvironment, we observed that OSCC has heterogeneous patterns of tumor-nerve interaction. Therefore, tumor cells could be found away from nerves, which was defined here as PNI type 0 (**Figures 1A, E**). The conditions that tumor cells encircling nerves <33% and ≥33% were separately divided into PNI type 1 (**Figures 1B, F**) and type 2 (**Figures 1C, G**). Once tumor cells were observed infiltrating into nerve sheathes, this pattern was defined as PNI type 3 (**Figures 1D, H**). Importantly, if more than one type of PNI simultaneously occurred in one tumor sample, this condition was concluded into the mixed PNI type. As the PNI type 0 could be present in all OSCC samples, its coexistence with other types was not considered mixed PNI types. Thus, there were 94 (51.4%), 17 (9.3%), 31 (16.9%), and 16 (8.7%) patients with single type of PNI, types 0–3, respectively. In addition, the remaining 25 (13.7%) patients had the mixed PNI type (**Figure 1I**).

### Mixed Types of PNI Are Present in One OSCC Patient

**Figure 2:**
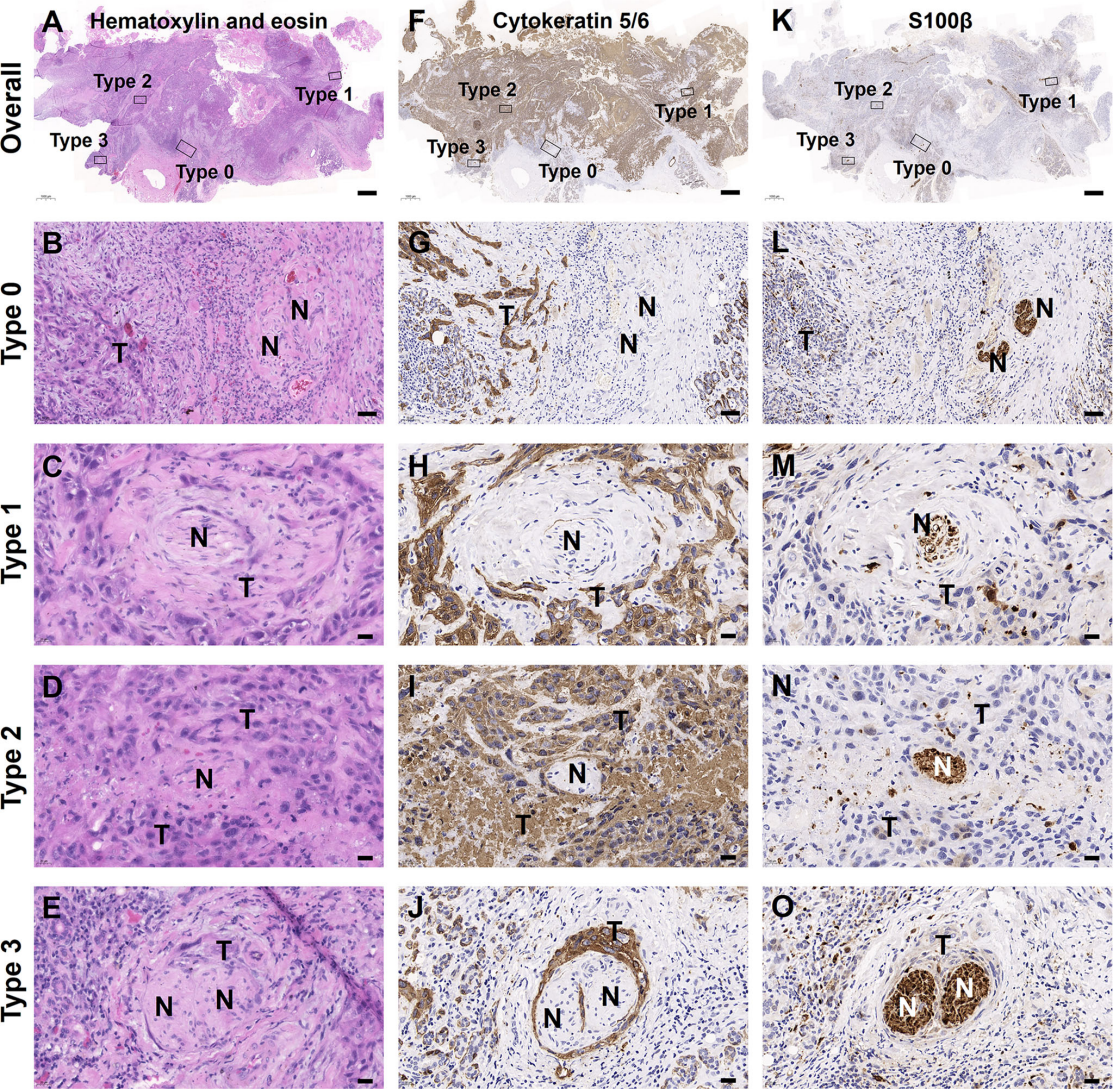
Mixed PNI types simultaneously occurred in one OSCC sample. **(A**–**E)** H&E images. **(F**–**J)** Tumor cells labeled with cytokeratin 5/6. **(K**–**O)** Nerves labeled with S100β. The bold “T” and “N” represented tumor cells and nerves, respectively. Scale bars: **(A**, **F**, **K)** 1,000 μm, **(B**, **G**, **L)** 50 μm, and **(C**–**E**, **H**–**J**, **M**–**O)** 20 μm.

In this study, we found that PNI^−^ status contained two patterns of tumor-nerve interaction, that is PNI types 0 and 1, while PNI^+^ status consisted of PNI types 2 and 3. The traditional PNI^−^ patients did not contain any mixed types of PNI. However, in the PNI^+^ patients, the mixed types of PNI consisted of type 1/2 (6, 3.3%), type 2/3 (13, 7.1%), type 1/3 (5, 2.7%), and type 1/2/3 (1, 0.5%) (**Figure 1I**). Through immunohistochemical staining on sequential tissue sections, we showed that PNI types 0–3 simultaneously occurred in the same one OSCC sample (**Figure 2**).

### The PNI Status of OSCC Patients Should Be Subdivided Into Three Types of WPNI

**Figure 3:**
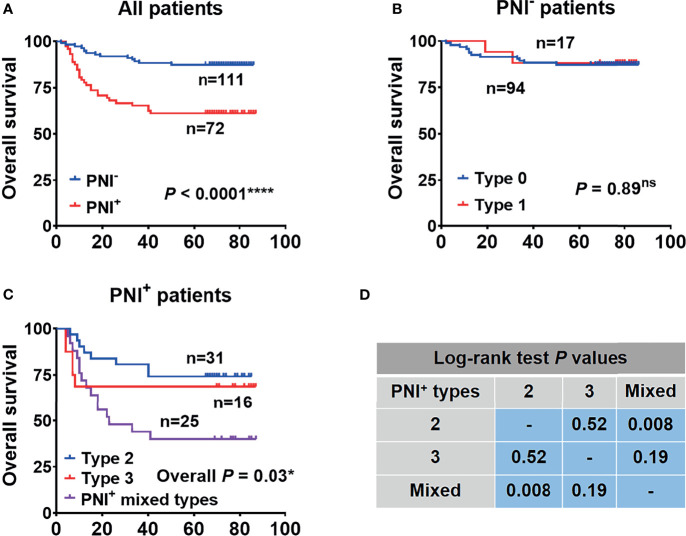
The overall survival analysis performed between patients with the traditional PNI by dichotomy **(A)** and subgroup analysis separately in PNI^−^ **(B)** and PNI^+^ **(C)** patients. **(D)** The results of log-rank test in **(C)**. *p<0.05; ****p<0.0001; ns, not significant.

In order to determine the prognostic value of PNI and its subtypes in our cohort, Kaplan-Meier analyses were firstly performed between the traditional PNI^+^ and PNI^−^ OSCC patients. Patients with the traditional PNI^+^ status showed a significantly lower OS than the PNI^−^ patients (*p* < 0.0001) (**Figure 3A**). Next, we did subgroup analysis and found that in the PNI^−^ patients, PNI types 0 and 1 both indicated high 5-year OS (87.2% *vs*. 88.2%, respectively; *p* = 0.89) (**Figure 3B**). However, in the PNI^+^ patients, PNI type 3 tended to indicate a decreased 5-year OS than PNI type 2 (68.8% *vs*. 74.2%, respectively) though statistically not significant (*p* = 0.52) (**Figures 3C, D**). Most importantly, patients with the mixed PNI types had the worst 5-year OS (40.0%) (**Figure 3C**).

**Figure 4:**
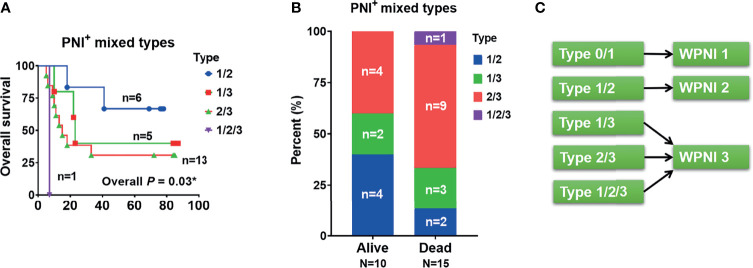
The PNI status of OSCC patients should be subdivided into three types of WPNI. **(A)** The overall survival curves were drawn and compared within PNI^+^ patients with mixed PNI types. **(B)** The stack graph showing the frequency distribution in PNI^+^ patients with mixed PNI types. **(C)** As the highest PNI type perfectly indicated the patients’ survival outcome, PNI type 0 or 1 was redefined as WPNI 1, the mixed PNI type 1/2 was reclassified as WPNI 2, and the mixed PNI types 1/3, 2/3, and 1/2/3 were combined as WPNI 3. *p<0.05.

In order to determine the contribution of PNI subtypes to the decrease of OS, we further drew and compared the OS curves within the mixed PNI types. As a result, PNI type 1/2 showed the best OS, followed by PNI types 1/3, 2/3, and 1/2/3 (**Figure 4A**). In detail, the proportion of deaths tended to increase from 13% (*n* = 2, PNI type 1/2) to 20% (*n* = 3, PNI type 1/3), and then to 60% (*n* = 9, PNI type 2/3) (**Figure 4B**). Based on the OS analysis above, the highest PNI type perfectly indicated the patients’ survival outcome. Thus, we here introduced the variable WPNI, which took the highest score present in each patient, to define the PNI status (**Figure 4C**). Thus, as PNI type 0 or 1 was redefined as WPNI 1, mixed PNI types of 1/2 were reclassified as WPNI 2. Meanwhile, we took the mixed PNI types 1/3, 2/3, and 1/2/3 as WPNI 3.

### WPNI 3 OSCC Patients Showed the Worst Clinical Outcome, Prognosis, and Immune Response

To investigate the correlation between the clinicopathological features and the new WPNI scoring system, 111 (60.7%), 37 (20.2%), and 35 (19.1%) OSCC patients were reclassified into WPNI 1, WPNI 2, and WPNI 3, respectively (**Figure 1I**). Chi-square test was performed and presented that higher WPNI indicated enhanced tumor lymph node metastasis (LN metastasis, *χ* ^2^ = 15.96, *p* < 0.001) and more aggressive pattern of tumor invasion (WPOI, *χ* ^2^ = 16.27, *p* < 0.001) (**Table 2**). Moreover, the rate of local-regional recurrence and distant metastasis after OSCC operation also significantly increased with higher WPNI score (**Table 2**).

**Table 2:** Clinicopathological features and their associations with the WPNI scoring system.

Features	WPNI			*χ* ^2^	*p*-value
	1 (*n* = 111)	2 (*n* = 37)	3 (*n* = 35)		
Sex				2.48	0.29
Female	52 (46.8%)	12 (32.4%)	14 (40.0%)		
Male	59 (53.2%)	25 (67.6%)	21 (60.0%)		
Age				1.13	0.57
≤60	53 (47.7%)	17 (45.9%)	20 (57.1%)		
>60	58 (52.3%)	20 (54.1%)	15 (42.9%)		
Tumor site				6.01	0.42
Buccal mucosa	21 (18.9%)	7 (18.9%)	5 (14.3%)		
Tongue	48 (43.2%)	17 (45.9%)	19 (54.3%)		
Gingiva	24 (21.6%)	3 (8.1%)	6 (17.1%)		
Others	18 (16.2%)	10 (27.0%)	5 (14.3%)		
Pathologic T				2.59	0.29
T1+T2	97 (87.4%)	33 (89.2%)	27 (77.1%)		
T3+T4	14 (12.6%)	4 (10.8%)	8 (22.9%)		
Pathologic N				15.96	<0.001^***^
N0	84 (75.7%)	16 (43.2%)	18 (51.4%)		
N1+N2	27 (24.3%)	21 (56.8%)	17 (48.6%)		
Tumor TNM stage				12.36	0.002^**^
I+II	74 (66.7%)	15 (40.5%)	14 (40.0%)		
III+IV	37 (33.3%)	22 (59.5%)	21 (60.0%)		
Tumor differentiation				3.73	0.15
Well	102 (91.9%)	33 (89.2%)	28 (80.0%)		
Moderately/Poor	9 (8.1%)	4 (10.8%)	7 (20.0%)		
Worst pattern of invasion				16.27	<0.001^***^
1–3	70 (63.1%)	16 (43.2%)	9 (25.7%)		
4–5	41 (36.9%)	21 (56.8%)	26 (74.3%)		
Radiotherapy				7.70	0.02^*^
Without	78 (70.3%)	18 (48.6%)	18 (51.4%)		
With	33 (29.7%)	19 (51.4%)	17 (48.6%)		
Chemotherapy				2.20	0.32
Without	97 (87.4%)	34 (91.9%)	28 (80.0%)		
With	14 (12.6%)	3 (8.1%)	7 (20%)		
Local-regional recurrence				21.81	<0.001^***^
No	98 (88.3%)	28 (75.7%)	18 (51.4%)		
Yes	13 (11.7%)	9 (24.3%)	17 (48.6%)		
Distant metastasis				12.78	<0.001^***^
No	105 (94.6%)	32 (86.5%)	25 (71.4%)		
Yes	6 (5.4%)	5 (13.5%)	10 (28.6%)		

WPNI, worst pattern of perineural invasion; TNM, tumor-node-metastasis. *p<0.05; **p<0.01; ***p<0.001.

**Figure 5:**
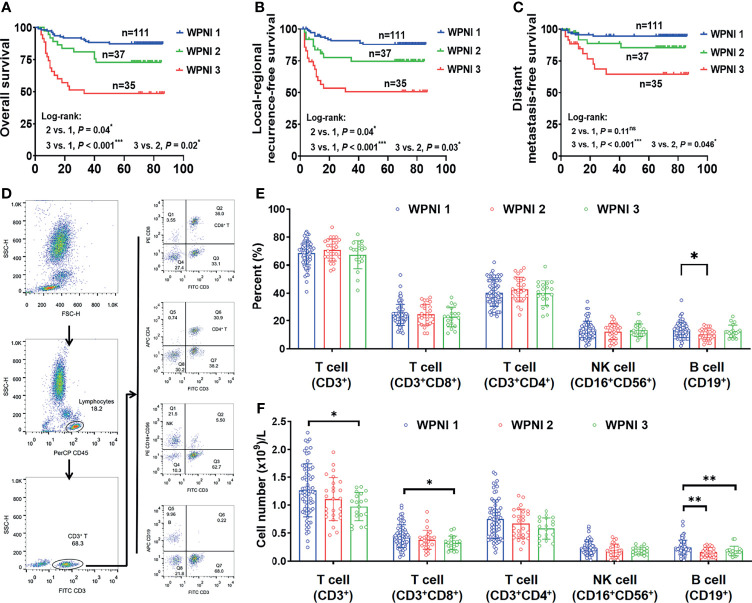
WPNI 3 OSCC patients showed the worst prognosis and immune response. **(A**–**C)** Kaplan-Meier analysis for overall survival **(A)**, local-regional recurrence-free survival **(B)**, and distant metastasis-free survival **(C)** in patients stratified by the WPNI scoring system. **(D)** The gating strategy for flow cytometry analysis. **(E, F)** Comparison of the circulating lymphocytes among OSCC patients with different WPNI scores.

To evaluate the prognostic value of the WPNI model on OS, LRFS, and DMFS, Kaplan-Meier analysis and log-rank test were performed. In all three survival models, WPNI 1 indicated the best prognosis while WPNI 3 predicated the worst prognosis (**Figures 5A–C**). As for WPNI 2, it had significantly better OS (*p* = 0.02), LRFS (*p* = 0.03), and DMFS (*p* = 0.046) than WPNI 3, which both indicated the traditionally PNI^+^ status. Furthermore, univariate Cox analysis showed that pathologic N stage, TNM stage, and WPNI were significantly negative predictors for OS, LRFS, and DMFS (**Table 3**). Tumor differentiation and WPOI could successfully predict OS and LRFS, but not DMFS (**Table 3**). To exclude the effects of confounders, multivariate Cox analysis was also performed that WPNI 3 was the only variable to independently predict OS (HR = 3.80, 95% CI = 1.83–7.86, *p* < 0.001), LRFS (HR = 3.85, 95% CI = 1.81–8.18, *p* < 0.001), and DMFS (HR = 5.29, 95% CI = 1.83–15.28, *p* = 0.002) (**Table 3**).

**Table 3:** Univariate and multivariate analysis for OS, LRFS, and DMFS.

Features	Univariate analysis			Multivariate analysis		
	HR	95%CI	*p*-value	HR	95%CI	*p*-value
**OS**						
Pathologic N stage (N1+N2 *vs*. N0)	6.89	3.46–13.74	<0.001^***^	4.47	1.00–20.00	0.050
Tumor TNM stage (III+IV *vs*. I+II)	6.03	2.88–12.61	<0.001^***^	1.27	0.26–6.21	0.766
Tumor differentiation (moderately/poor *vs*. well)	3.00	1.47–6.11	0.002^**^	1.73	0.50–3.71	0.161
WPOI (4–5 *vs*. 1–3)	2.70	1.40–5.19	0.003^**^	1.42	0.70–2.89	0.337
Chemotherapy (with *vs*. without)	2.41	1.18–4.90	0.015^*^	1.19	0.57–2.50	0.637
WPNI (2 *vs*. 1)	2.27	1.01–5.10	0.048^*^	1.32	0.57–3.06	0.513
WPNI (3 *vs*. 1)	5.72	2.84–11.53	<0.001^***^	3.80	1.83–7.86	<0.001^***^
**LRFS**						
Pathologic N stage (N1+N2 *vs*. N0)	3.72	1.95–7.11	<0.001^***^	2.59	0.57–11.75	0.219
Tumor TNM stage (III+IV *vs*. I+II)	3.13	1.60–6.09	0.001^**^	1.10	0.23–5.17	0.907
Tumor differentiation (moderately/poor *vs*. well)	2.40	1.10–5.23	0.028^*^	1.29	0.56–2.93	0.550
WPOI (4–5 *vs*. 1–3)	3.54	1.72–7.27	0.001^**^	2.17	1.00–4.72	0.051
WPNI (2 *vs*. 1)	2.33	1.00–5.46	0.051	1.47	0.61–3.53	0.387
WPNI (3 *vs*. 1)	5.67	2.75–11.69	<0.001^***^	3.85	1.81–8.18	<0.001^***^
**DMFS**						
Sex (male *vs*. female)	3.12	1.05–9.29	0.040^*^	2.50	0.83–7.58	0.104
Pathologic N stage (N1+N2 *vs*. N0)	3.80	1.57–9.19	0.003^**^	3.04	0.38–24.05	0.292
Tumor TNM stage (III+IV *vs*. I+II)	3.15	1.27–7.82	0.013^*^	0.89	0.11–7.38	0.912
WPNI (2 *vs*. 1)	2.60	0.79–8.51	0.115	1.58	0.45–5.47	0.474
WPNI (3 *vs*. 1)	7.01	2.54–19.38	<0.001^***^	5.29	1.83–15.28	0.002^**^

OS, overall survival; LRFS, local-regional recurrence-free survival; DMFS, distant metastasis-free survival; HR, hazard ratio; 95% CI, 95% confidence interval; TNM, tumor-node-metastasis; WPOI, worst pattern of invasion; WPNI, worst pattern of perineural invasion. *p<0.05; **p<0.01; ***p<0.001.

As reported that tumor-infiltrating lymphocytes significantly correlated with PNI (25, 26), we further explored whether OSCC patients with different WPNI scores had changed lymphocyte subsets in the preoperative peripheral blood. The gating strategy for grouping cell populations is shown in **Figure 5D**. Although the proportion of lymphocyte subsets did not significantly change with different WPNI scores (**Figure 5E**), the absolute number of total T cells (CD3^+^), inhibitory T cells (CD3^+^CD8^+^), and B cells (CD19^+^) were all significantly decreased in WPNI 3 patients (*p* = 0.01, *p* = 0.01, and *p* = 0.008, respectively; **Figure 5F**), further suggesting a possible imbalance in immune response.

## Discussion

Classical PNI types in different studies showed inconsistent prognostic value. Thus, alternative PNI-related characteristics were developed to test their rationality. Karina et al. retrospectively analyzed the prognostic value of PNI, number of PNIs, distance between PNI and cancer center, and diameter of invaded nerves in PNI using 318 OSCC samples and showed that classical PNI classification (PNI^−^ *vs*. PNI^+^) could not predict the prognosis successfully (27). In contrast, an increased number of PNIs could independently predict a higher risk of local recurrence, and the prognosis worsened when the diameter of the invaded nerves exceeded 1 mm. In addition, a single PNI or PNI occurring outside the cancer center had no effect on the prognosis. Wei et al. also found that with an increased number of PNIs, the prognosis of patients worsened, but the author pointed out that how to quantify the number of PNIs needs further study (28). Miller et al. classified PNI into “PNI in tumor center,” “PNI at tumor border,” “PNI outside tumor center,” and “no PNI” in head and neck cancer and found that this classification system could not effectively predict local recurrence (29). However, in 200 patients with oral tongue SCC, Caponio et al. presented that intratumoral PNI combined with tumor grading and WPOI could successfully predict lymph-node metastasis and advocated integrating PNI in the 8th edition of the AJCC Cancer staging system (30).

Given that PNI reflects the interaction between tumor cells and nerves (31), and it has been proven to be an active process (15), tumor cells “away from” nerves to “encircling” them and finally “infiltrating into” nerve sheathes can coexist in one tumor. Notably, tumor cells encircling nerves were empirically divided into two conditions based on the cutoff value of 33% which was empirically adopted (16). Moreover, intratumoral heterogeneity might be the intrinsic driver for the simultaneous existence of several PNI types within a single tumor microenvironment (TME). For the first time, we classified the traditional PNI^−^ status into WPNI 1, which represented an early stage of tumor cells invading nerves. Survival analysis confirmed that OSCC patients with WPNI 1 did have a good prognosis. However, the traditional PNI^+^ indicator should be critically evaluated for its further subclassification. In this study, PNI^+^ successfully predicated worse survival, but we found that WPNI 2 had significantly better survival than WPNI 3. We believed that as the late stage of tumor cells invading nerves, WPNI 3 contributed mainly to the predictive ability of the traditionally PNI^+^ status. Multivariate Cox analysis confirmed that only WPNI 3 could independently predict the patients’ survival. So, tumor cell-mediated destruction of nerve sheaths and their subsequent invasion significantly accelerate tumor progression. Once tumor cells entered the nerve microenvironment, they would have convenient routes and abundant nutrition for their distant metastasis (32).

Inflammatory infiltration related to PNI has been intensively investigated (33–35); we here focused on how the circulating lymphocytes changed with different stages of tumor cells invading nerves. In pancreatic ductal adenocarcinomas, infiltrating CD8^+^ T cells significantly decreased in the PNI^+^ samples accompanied with elevated levels of acetylcholine (25). In OSCCs, higher CD8^+^ T cells at the parenchyma of the invading edge and peripheral stroma both indicated improved overall and recurrence-free survival (36). In our study, WPNI 3, but not WPNI 2, represented significantly lower circulating CD8^+^ T cells than WPNI 1. Most importantly, WPNI 3 implies the destructed nerve sheathes, which means more possibility for neurotransmitters such as norepinephrine spilling into OSCC microenvironment (37). What is more, B cells also decreased as WPNI scores increase, indicating the damaged immune response.

In conclusion, PNI tends to be an active and continuous process in which tumor cells move far away from nerves to invade the nerve sheaths, which reflects clinically worsening survival. Therefore, the WPNI scoring system, which takes the highest score to refine the traditional PNI status, may be worth further clinical evaluation and promotion.

## Data Availability Statement

The original contributions presented in the study are included in the article/**Supplementary Material**. Further inquiries can be directed to the corresponding authors.

## Ethics Statement

The studies involving human participants were reviewed and approved by The Research Ethics Committee of Nanjing Stomatological Hospital. The patients/participants provided their written informed consent to participate in this study.

## Author Contributions

QH, YN, and LD designed this study. YF and XWZ performed all the experiments. YF, XWZ, and ZD collected clinical data. NZ, YS, and XXZ interpreted the data. YJ, YY, XH, and LZ offered technical support. YF and XWZ wrote the manuscript. All authors contributed to the article and approved the submitted version.

## Funding

This work was supported by the National Natural Science Foundation of China (81902754, 81702680, 82002865, 81772880), Natural Science Foundation of Jiangsu Province (BK20190304, BE2020628), and Nanjing Medical Science and Technology Development Foundation (YKK19091, YKK20153).

## Conflict of Interest

The authors declare that the research was conducted in the absence of any commercial or financial relationships that could be construed as a potential conflict of interest.

## Publisher’s Note

All claims expressed in this article are solely those of the authors and do not necessarily represent those of their affiliated organizations, or those of the publisher, the editors and the reviewers. Any product that may be evaluated in this article, or claim that may be made by its manufacturer, is not guaranteed or endorsed by the publisher.
