# Supplementary material for: Worst Pattern of Perineural Invasion Redefines the Spatial Localization of Nerves in Oral Squamous Cell Carcinoma
Source: Front Oncol. 2021 Nov 29;11:766902. doi: 10.3389/fonc.2021.766902 (PMC8667170; doi:10.3389/fonc.2021.766902)
Supplement: Supplementary file 1 [file Table_1.docx]

Supplementary Table 1. The clinicopathological features of 114 OSCC patients with blood data

| **Features** | **n (%)** |
| --- | --- |
| Age (years): Median (Range) | 56 (47-82) |
| Sex |  |
| Female | 49 (43.0) |
| Male | 65 (57.0) |
| Tumour Site |  |
| Buccal mucosa | 22 (19.3) |
| Tongue | 55 (48.2) |
| Gingiva | 18 (15.8) |
| Others | 19 (16.7) |
| Pathologic T stage |  |
| T1 | 35 (30.7) |
| T2 | 60 (52.6) |
| T3 | 8 (7.0) |
| T4 | 11 (9.6) |
| Pathologic N stage |  |
| N0 | 67 (58.8) |
| N1 | 29 (25.4) |
| N2 | 18 (15.8) |
| Tumour TNM Stage |  |
| I | 21 (18.4) |
| II | 35 (30.7) |
| III | 33 (28.9) |
| IV | 25 (21.9) |
| Tumour differentiation |  |
| Well | 102 (89.5) |
| Moderately/Poor | 12 (10.5) |
| Worst pattern of invasion (WPOI) |  |
| 1-3 | 59 (51.8) |
| 4-5 | 55 (48.2) |
| Radiotherapy |  |
| Without | 64 (56.1) |
| With | 50 (43.9) |
| Chemotherapy |  |
| Without | 99 (86.8) |
| With | 15 (13.2) |
